# 2018 *PLOS Genetics* Research Prize: Bundling, stabilizing, organizing—The orchestration of acentriolar spindle assembly by microtubule motor proteins

**DOI:** 10.1371/journal.pgen.1007649

**Published:** 2018-09-13

**Authors:** Gregory S. Barsh, Needhi Bhalla, Francesca Cole, Gregory P. Copenhaver, Soni Lacefield, Diana E. Libuda

**Affiliations:** 1 HudsonAlpha Institute for Biotechnology, Huntsville, Alabama, United States of America; 2 Department of Genetics, Stanford University School of Medicine, Stanford, California, United States of America; 3 Department of Molecular, Cell and Developmental Biology, University of California, Santa Cruz, Santa Cruz, California, United States of America; 4 Department of Epigenetics and Molecular Carcinogenesis, University of Texas MD Anderson Cancer Center, Smithville, Texas, United States of America; 5 Department of Biology and the Integrative Program for Biological and Genome Sciences, University of North Carolina at Chapel Hill, Chapel Hill, North Carolina, United States of America; 6 Department of Biology, Indiana University, Bloomington, Indiana, United States of America; 7 Department of Biology, Institute of Molecular Biology, University of Oregon, Eugene, Oregon, United States of America

*PLOS Genetics* is a community journal: run by working scientists, for working scientists. One of the most rewarding aspects of serving as an editor is the opportunity to see, appreciate, and celebrate great science from our authors. With this motivation in mind, the annual *PLOS Genetics* Research Prize was established several years ago to recognize a paper published in the previous 12 months that was scientifically excellent and had broad impact across the genetics community. Nominations are open to the public, and the winner is selected by the *PLOS Genetics* Editors-in-Chief and Section Editors. This year, there were a number of very strong nominations. Besides the prize recipient described further below, there are two additional papers that are especially notable. An article by Amelie Baud and colleagues demonstrates that over 100 diverse phenotypes in mice are affected by social interactions [1]. This work was fascinating because it examines a relatively under-studied phenomenon that has far-reaching implications for genetic analyses. It also received broad attention with coverage in over 39 media reports and blogs. Another article by Carlos Eduardo Amorim and colleagues examined the long-standing quandary of why lethal alleles persist in human populations, and comes to the surprising conclusion that ascertainment bias is a significant contributing factor [2]. This work was also broadly impactful and widely discussed on social media.

This year’s prize recipient is an article by Timothy Mullen and Sarah Wignall [3], striking in many ways, not the least of which was that it was nominated independently by four different members of the genetics community. In what follows, these nominators tell us more about the significance and impact of the work.

During chromosome segregation, the spindle is assembled from microtubules to accurately partition chromosomes. In most systems, spindle assembly initiates from centriole-containing centrosomes, generating a highly organized, polarized array of microtubules capable of pulling chromosomes to opposite poles [4]. However, female reproductive cells (oocytes) in many species, including humans, segregate chromosomes on acentriolar spindles assembled through unique mechanisms. Acentriolar spindles are frequently associated with aberrant chromosome segregation in cancer cells, raising the question of how and why oocytes rely upon a potentially less reliable spindle for segregation. Identifying and characterizing how oocytes address the considerable challenge of assembling a bipolar, highly organized array of microtubules in the absence of centrioles is a major open question in the field of chromosome segregation.

The small nematode worm, *C*. *elegans*, provides a genetically and visually tractable system to interrogate the control of meiotic chromosome segregation during oogenesis. Several labs have contributed to our understanding of spindle assembly during *C*. *elegans* oogenesis (see review in [5], e.g., [6–11]). These studies have identified activities that sort and organize microtubules to promote spindle bipolarity, factors that bundle microtubules to generate a durable structure, and proteins that stabilize microtubules to promote both proper chromosome alignment during metaphase and segregation during anaphase. However, how these individual behaviors are coordinated with one another and with progression through the meiotic cell cycle is unknown.

Meiotic acentriolar spindle assembly in *C*. *elegans* occurs in a progressive fashion. After nuclear envelope breakdown, microtubules bundle to form a cage-like structure that surrounds meiotic chromosomes [10]. The microtubules are sorted so that multipolar spindles, with minus ends at the periphery, are visible. These multiple poles subsequently coalesce into the stereotypical bipolar structure in metaphase and segregate chromosomes during anaphase [7]. Mullen and Wignall identified two redundant members of a conserved family of minus-end directed motor proteins, KLP-15 and KLP-16, that were required early in the acentriolar spindle assembly process [1]. In their absence, microtubules assemble into the cage-like structure but fail to stabilize and bundle, resulting in a collapsed, disorganized spindle. Remarkably, loss of KLP-15 and KLP-16 results in collapsed spindles only during acentriolar spindle formation and not during the centriolar spindle formation of mitotic divisions, suggesting that these motor proteins are only essential in cells that lack centrioles.

An obvious prediction of these experiments might be that the collapsed spindles cannot support chromosome segregation. However, Mullen and Wignall were surprised to observe that despite substantial defects in spindle assembly when KLP-15/16 were knocked down, oocytes were able to eventually bundle and organize microtubules to segregate chromosomes in anaphase, albeit inaccurately. These thorough and meticulous observations suggested that other, redundant mechanisms can drive microtubule bundling and chromosome segregation in anaphase. After screening several candidates based on their mitotic centriolar spindle function and localization along the meiotic spindle, the authors discovered previously unknown roles for the microtubule bundling protein SPD-1 and the kinesin-12 family motor protein KLP-18. Specifically, in the absence of KLP-15/16, SPD-1 bundled and KLP-18 organized microtubules later during anaphase to support chromosome segregation. While a similar sorting activity had previously been assigned to KLP-18 during spindle assembly [7, 10, 12], a role in sorting during anaphase could only be detected in a sensitized KLP-15/16 knockdown system. Similarly, a role for SPD-1 in acentriolar chromosome segregation could not be appreciated when KLP-15 and KLP-16 successfully promote microtubule bundling during spindle assembly.

Overall, by carefully analyzing their phenotypes, the results from Mullen and Wignall revealed that multiple microtubule motor proteins coordinate their activity to promote acentriolar spindle assembly and chromosome segregation during oocyte meiosis. These elegant experiments highlight two important lessons in biological research: 1) the requirement for individual factors to coordinate their specific activities to generate large macromolecular machines, such as the spindle, and 2) the contribution of redundancy to promote the robustness of complex cell behaviors.

## References

[1] Baud A, Mulligan MK, Casale FP, Ingels JF, Bohl CJ, Callebert J, et al Genetic Variation in the Social Environment Contributes to Health and Disease. PLOS Genet. 2017; 13(1): e1006498 10.1371/journal.pgen.1006498 28121987 PMC5266220

[2] Amorim CEG, Gao Z, Baker Z, Diesel JF, Simons YB, Haque IS, et al The population genetics of human disease: The case of recessive, lethal mutations. PLOS Genet. 2017; 13(9): e1006915 10.1371/journal.pgen.1006915 28957316 PMC5619689

[3] Mullen TJ, Wignall SM. Interplay between microtubule bundling and sorting factors ensures acentriolar spindle stability during C. elegans oocyte meiosis. PLOS Genet. 2017; 13(9): e1006986 10.1371/journal.pgen.1006986 28910277 PMC5614648

[4] Dumont J, Desai A. Acentrosomal spindle assembly and chromosome segregation during oocyte meiosis. Trends Cell Biol. 2012;22(5):241–9. 10.1016/j.tcb.2012.02.007. 22480579 PMC3348331

[5] Severson AF, von Dassow G, Bowerman B. Oocyte Meiotic Spindle Assembly and Function. Curr Top Dev Biol. 2016;116:65–98. Epub 2016/03/13. 10.1016/bs.ctdb.2015.11.031. 26970614 PMC7744206

[6] Albertson DG, Thomson JN. Segregation of holocentric chromosomes at meiosis in the nematode, Caenorhabditis elegans. Chromosome Res. 1993;1(1):15–26. . 8143084 10.1007/BF00710603

[7] Wignall SM, Villeneuve AM. Lateral microtubule bundles promote chromosome alignment during acentrosomal oocyte meiosis. Nature cell biology. 2009;11(7):839–44. 10.1038/ncb1891 19525937 PMC2760407

[8] McNally KP, Panzica MT, Kim T, Cortes DB, McNally FJ. A novel chromosome segregation mechanism during female meiosis. Mol Biol Cell. 2016;27(16):2576–89. 10.1091/mbc.e16-05-0331. 27335123 PMC4985259

[9] Dumont J, Oegema K, Desai A. A kinetochore-independent mechanism drives anaphase chromosome separation during acentrosomal meiosis. Nature cell biology. 2010;12(9):894–901. 10.1038/ncb2093 20729837 PMC3052858

[10] Wolff ID, Tran MV, Mullen TJ, Villeneuve AM, Wignall SM. Assembly of Caenorhabditis elegans acentrosomal spindles occurs without evident microtubule-organizing centers and requires microtubule sorting by KLP-18/kinesin-12 and MESP-1. Mol Biol Cell. 2016;27(20):3122–31. 10.1091/mbc.e16-05-0291. 27559133 PMC5063619

[11] Connolly AA, Osterberg V, Christensen S, Price M, Lu C, Chicas-Cruz K, et al Caenorhabditis elegans oocyte meiotic spindle pole assembly requires microtubule severing and the calponin homology domain protein ASPM-1. Mol Biol Cell. 2014;25(8):1298–311. 10.1091/mbc.e13-11-0687. 24554763 PMC3982995

[12] Segbert C, Barkus R, Powers J, Strome S, Saxton WM, Bossinger O. KLP-18, a Klp2 kinesin, is required for assembly of acentrosomal meiotic spindles in Caenorhabditis elegans. Mol Biol Cell. 2003;14(11):4458–69. 10.1091/mbc.e03-05-0283. 12937278 PMC266765

